# Monogamy in a Moment: How do Brief Social Interactions Change Over Time in Pair-Bonded Zebra Finches (*Taeniopygia guttata*)?

**DOI:** 10.1093/iob/obaa034

**Published:** 2020-12-26

**Authors:** Nora H Prior, Edward Smith, Robert J Dooling, Gregory F Ball

**Affiliations:** Department of Psychology, University of Maryland, College Park, MD, USA

## Abstract

Research on monogamy has largely focused on marked behaviors that are unique to pair bonded partners. However, these marked behaviors represent only a subset of the pair-directed behaviors that partners engage in; the influence of pair bonding on mundane or subtle social interactions among partners remains largely unknown. In this study, we describe the changes that occur during brief social reunions (or greets) over the course of pair bonding in zebra finches. We quantified pair-directed behavior during 5-min reunions from three stages of pair bonding: initial pairing (between 4 and 72 h), early pairing (1–2 weeks), and late pairing (>1 month). These social interactions were operationalized in multiple ways. First, we quantified the overall activity levels (call and movement rates) for both the male and female. Overall, females were more active than males, but for both males and females calling activity was highest at initial pairing. We quantified behavioral coordination between partners in two ways: (1) similarity in call and movement rates between partners and (2) temporal synchrony of calls and movements between partners (via sliding correlation coefficients of time-stamped calls and movements). Overall, there were no effects of pairing stage on behavioral coordination. Finally, we used principal component analyses to disentangle behavioral coordination from the activity levels of the male and female. These results contribute to a growing line of evidence that male and female zebra finches differentially contribute to social dynamics and highlight the influence of pair bonding on the development of social dynamics. Furthermore, our preliminary analyses raise the hypothesis that behavioral coordination during the earliest phases of pairing is modulated by the extent and nature of prior experience. Overall, while behavioral coordination is clearly important for many salient interactions such as duetting, courtship displays, and biparental care, the significance of mundane social interactions for monogamous partnerships remains largely unknown.

## Introduction

In monogamous species, the formation and maintenance of a pair bond are necessary for the successful rearing of offspring (Lack 1968; Reichard and Boesch 2003). The majority of research on the mechanisms underlying pair bonding has focused on marked, pair-specific behaviors, and interactions between partners (Wachtmeister 2001; Young and Wang 2004; Aragona et al. 2006; Young et al. 2011; Soma and Garamszegi 2015). This approach has revealed remarkable conservation in the mechanisms of pair bonding across taxa (Bales et al. 2007; O’Connell and Hofmann 2012; Donaldson and Young 2016; Walum and Young 2018).

Many pairing behaviors are visible, or marked, and these behaviors and interactions are particularly evident during courtship displays and the rapid changes in pair-directed behavior during pair bond formation. For example, in prairie voles (*Microtus ochrogaster*), the establishment of a pair bond is clearly marked by the development of selective preference/affiliation for a mate (Williams et al. 1992; Young et al. 2011; Resendez et al. 2016). However, these marked behaviors represent only a subset of those that partners engage in, and more detailed understandings of partner interactions are needed in order to further elucidate mechanisms of pair bonding. For example, considering prairie voles, the pattern of approach and proximity time between partners assayed during partner preference tests continues to change even after initial establishment of the pair bond. During later stages of pairing, individuals tend to spend more time with their partners (Scribner et al. 2020). Furthermore, important long-term impacts of pair bonding on the brain and behavior of voles are revealed by differences in approach of an individual to its mate versus a stranger (Scribner et al. 2020). This highlights how even in the most commonly studied model systems, remarkably little is known about the consequences of pair bonding on mundane or subtle features of partner interactions.

Whereas monogamy is rare in mammals (<4% of species), the vast majority of birds form some type of monogamous partnership (∼90% of species) (Reichard and Boesch 2003). Across bird species, there is considerable variation in the phenotype of monogamous bonds. Monogamous bonds vary in how long they are maintained; they may be transient, lasting only a season, or life-long (Black and Hulme 1996). As with prairie voles, the majority of avian studies focus on marked features of pair bonding (e.g., partner preference, proximity time, allopreening, and clumping) (Tomaszycki and Adkins-Regan 2005; Smiley et al. 2012; Prior et al. 2013; Kenny et al. 2017). However, the importance of brief social interactions has also been described in a wide range of species: at the nest, brief social interactions appear to be essential for the active coordination of parental duties between partners (Mariette and Griffith 2012, 2015; van Rooij and Griffith 2013; Boucaud et al. 2016b). This raises the question more broadly of how subtle features of social interactions are shaped by and contribute to long-term pair bond maintenance.

Here we describe the effect of pair bonding on brief social interactions in monogamous zebra finch pairs. Zebra finches maintain sexually monogamous life-long pair bonds, are nonterritorial, and are highly gregarious. Interestingly, traditional partner preference paradigms can fail to demonstrate selective preference for the partner (Prior et al. 2013), although other behavioral assays clearly show that many aspects of pair-directed behavior are reserved for or more common between partners than familiar conspecifics (Gill et al. 2015; Fernandez et al. 2017). Additionally, intra-pair calling dynamics, across multiple contexts, appear to be an important behavioral component of the zebra finch pair bond (Elie et al. 2010; Gill et al. 2015; Boucaud et al. 2016a; D’Amelio et al. 2017b; Fernandez et al. 2017).

In this study, we operationalize brief social reunions (focusing on both vocal behavior as well as physical movements) of pairs over the course of pair bonding: initial pairing (between 4 and 72 h), early pairing (1–2 weeks), and late pairing (>1 month). First, we quantified the overall activity levels (call and movement rates) for both males and females. Second, we used two approaches to estimate the coordination of the activity between partners, including quantifying the similarity in call and movement rates between partners as well as quantifying the sliding correlation coefficients for time-stamped calls and movements (a measure of temporal synchrony). Finally, we used principal component analyses (PCAs) to disentangle behavioral coordination from the activity levels of the male and female.

## Materials and methods

### Subjects and establishment of pairs

Twenty adult zebra finches (5–6 months old) were used in this study (10 females and 10 males). Throughout the study, zebra finches were housed with *ad libitum* seed, water and grit on a 12L:12D light cycle. This same cohort of zebra finches was also used for a subsequent experiment (Prior et al. 2020), and many of the methods are similar and previously described. For clarity, we summarize all components relevant to this study.

Prior to pairing, zebra finches were housed in same-sex flocks. In order to provide the opportunity for pairs to freely form, birds were moved to mixed-sex flocks for 72 h. Providing individuals with the opportunity to freely form monogamous bonds is important as forced pairing can be associated with lower pair fecundity (Griffith et al. 2017). Thus, birds were housed in mixed-sex flocks with either a male- or female-biased sex ratio (two females with three males or three females with two males). Pair bonding was assessed visually each day; occurrences of selective affiliative behaviors (i.e., clumping, allopreening, and coordinated preening) between individuals were scored during 5-min behavioral observations. After 72 h, birds were removed from mixed-sex flocks and housed with their mates for the duration of the study. Note that it is typical for not all birds in mixed-sex flocks to form pair bonds (Smiley et al. 2012; Tomaszycki and Zatirka 2014; Scalera and Tomaszycki 2018). Here we identified four clearly-established pairs (paired). Another four pairs were selected from the same mixed-sex flocks of birds that showed little evidence of pairing. The last two pairs were composed of birds unfamiliar with each other. Thus, we established 10 zebra finch pairs with varying extents and patterns of prior experience. We predicted that prior experience would affect behavioral coordination in our reunion paradigm. Thus, despite that there were few birds in each group, we used prior experience as a factor in our later analyses (Paired *N* = 4, Weakly Paired *N* = 4, Force Paired *N* = 2). Regardless of prior experience, we have several indications that all pairs were indeed pair bonded. All pairs were seen being highly affiliative in the home cage and were not seen interacting aggressively. Additionally, after this experiment, pairs were provided multiple opportunities to nest and breed. Ultimately, all pairs, including those force-paired, attempted to breed (nest building and/or egg laying) and 8 out of 10 pairs (7 out of 10 during the first opportunity provided) successfully fledged chicks.

### Experimental design

**Fig. 1:**
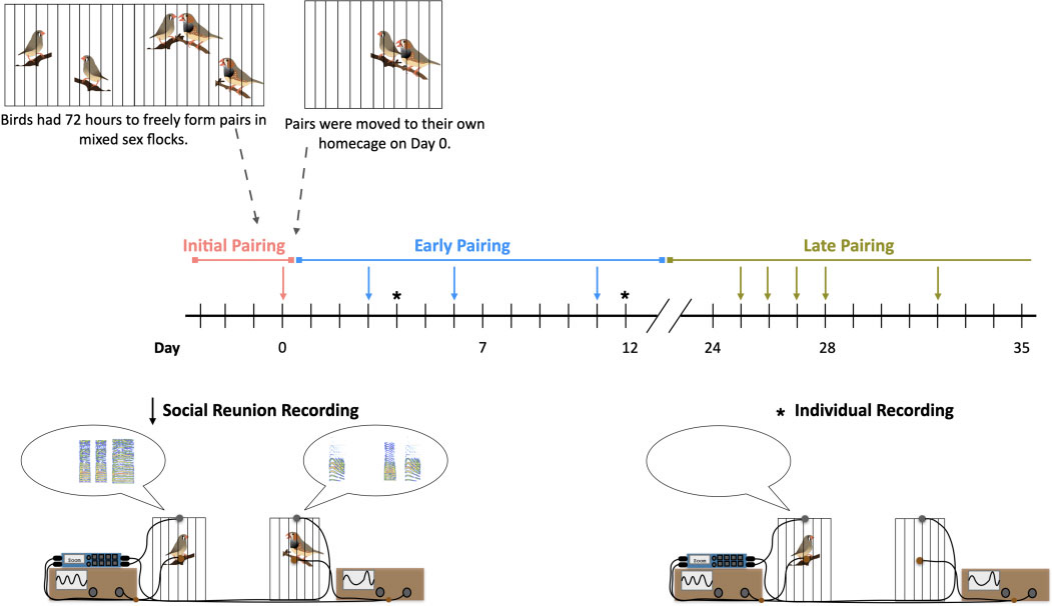
Diagram of the timeline of initial pair formation and behavioral recordings during the first month of pairing. All birds were given the opportunity to freely form a pair bond in mixed-sex flocks over the course of 72 h. The first social reunion was recorded on the day that pairs were moved from mixed-sex flocks to a new home cage with their partner (“initial pairing”). At this timepoint, pairs had been removed from the flock for 4–72 h. At the time of pairing, four pairs from the mixed-sex flocks clearly engaged in selective pairing behavior, four pairs were formed from birds that had been together in mixed-sex flocks but were not obviously pair bonded, and two pairs were composed of individuals that had not been in the same mixed-sex flocks and had no prior experience. We recorded reunion behavior from three timepoints during the following 2 weeks (“early pairing”), and from five timepoints during a late stage of pairing (>1-month post pairing) (“late pairing”*)*. Social reunion recording days are indicated on the timeline by an arrow*.* Additionally, as a control, two recordings were made of each individual alone in the testing room (individual recordings). Isolation recording days are indicated on the timeline by an asterisk. A schematic illustration of the social reunion behavior paradigm is shown on the bottom; redrawn from Prior et al. (2020). Briefly, behavior was recorded using four channels of a Zoom recorder (F8): movement was recorded from a piezo sensor attached to the perch of a smaller cage (indicated via oscilloscope), and acoustic behavior was recorded using tie clip microphones (indicated via spectrogram). Birds were also recorded in this set up alone (individual recording, shown bottom right).

A timeline of the behavioral recordings is presented in Fig. 1. We recorded brief social interactions from each pair 9 times over the course of the first month of pairing (note that we also recorded each individual twice in the room alone, see Fig. 1). These nine recordings were not evenly distributed over the course of the month, but were instead situated within periods of the pair bonding process commonly described in research. Pairing can be conceptually divided up into three stages: a brief courtship phase, a short pair formation phase, and an indefinite pair maintenance phase. Although these stages are commonly referenced (Smiley et al. 2012; Prior and Soma 2015; Resendez et al. 2016; Scribner et al. 2020), what distinguishes these stages and how long they last is unclear. In general, pair maintenance encompasses anything that occurs after the establishment of a pair bond. In zebra finches, pair bond formation can take up to 2 weeks; however, it is typically assumed to occur much more quickly (on the order of hours to days) (Zann 1996).

Here we recorded the first social reunion on the day that pairs were moved from mixed-sex flocks to a new home cage with their partner (“initial pairing”). At this timepoint, all pairs had been given the opportunity to engage in courtship behaviors and copulate; however, depending on how they were paired (“Prior Experience” described above), this time ranged from 4 to 72 h together. Thus, we might expect that a mix of courtship and pair formation could be occurring at this timepoint. Next, we recorded reunion behavior from three timepoints during the following 2 weeks (“early pairing”). By 2 weeks, we would expect all pair bonds to be established. The majority of research on pairing does not extend past 2–3 weeks post-pairing (Tomaszycki and Adkins-Regan 2005; Smiley et al. 2012; Tomaszycki and Zatirka 2014; Scalera and Tomaszycki 2018), and anything beyond this is typically considered pair maintenance (Tomaszycki and Adkins-Regan 2006; Prior et al. 2013; Scribner et al. 2020). We recorded reunion behavior five times during a late stage of pairing (>1 month post-pairing), which is unambiguously considered pair maintenance.

### Behavioral recordings

Recordings were made in a sound-attenuated test room, separate from the colony room. Partners were transported, one at a time, in a small covered cage and placed into individual cages each equipped with a tie-clip microphone and a piezo electric sensor attached to the perch (Fig. 1). The transport of each partner took 2–3 min, thus partners were separated for 4–6 min during transportation. This brief separation period is sufficient to elicit a social reunion (greeting behavior). Partners were not always transported in the same order (some days the male was transported first and other days the female was transported first). Upon placement of the second partner in the cage, reunion behavior was recorded. All four channels (one movement and one acoustic channel for each partner) were recorded using a multi-channel Zoom recorder (F8). Thus, we were able to make single recordings with temporally aligned, individually identifiable acoustic and movement behavior from each partner.

We have previously demonstrated that the majority of activity during this behavioral assay occurs within the first few minutes (<5 min) (Prior et al. 2020). Thus, only the first 5 min of the social reunion was analyzed. Although our behavioral assay clearly elicits socially-directed greeting/reunion behavior, we were concerned that such behavior during these brief behavioral assays would be easily influenced by other factors such as an individual’s experience immediately prior to the assay (events in the home cage prior to testing, and aspects of the transport). Therefore, we took several precautions to ensure repeatable conditions for quantification of behavior. First, we minimized the stress of handling by habituating birds to transportation prior to the start of the experiment. Additionally, once in the testing room, birds were not handled and were allowed to enter the testing cage/exit the transport cage on their own, with the researcher present. Second, we habituated birds to the behavioral procedure prior to the start of the experiment to ensure that any behavioral changes over the course of pairing were not simply caused by habituation to the paradigm. Habituation included 10 consecutive days of transport to the testing room (transport only = 4 days; testing room alone [individual recording] = 3 days; and social reunion with a same-sex flock mate = 3 days). The last day of habituation was the day before birds were moved to mixed-sex flocks. Third, we quantified behavior in our assay multiple times during each pairing stage (after the initial pairing). Finally, we included prior experience at the start of pairing as a factor in our analyses, as it may influence pairing dynamics.

### Scoring behavior during social reunions

#### Operational definitions of calls and movements

Overall movement and call rates were quantified for each partner during the first 5 min of the social reunion. Using an in-house MATLAB program (written by E.S.) we automatically identified time-stamped movements for each partner (Prior et al. 2020). Following initial observations and pilot experiments, we decided to group all movements together for three reasons. First, individuals produced a small repertoire of behaviors. All of the behaviors, we observed appeared to be related to the social interactions and included large body movements (perch hops) and small movements (including head tilts, fluff ups, and wing movements). Note that our goal was to reliably elicit social interactions, so the cages were small and contained no additional stimulation. Second, it was difficult to disentangle discrete movements (both during observations and from the recording on the time waveform) because large and small movements often occurred simultaneously or happened in rapid succession. Finally, we had no *a priori* reason to differentiate between large and small body movements.

Calls were semi-automatically classified to identify time-stamped calls for each partner (in-house MATLAB program written by E.S.). An initial automatic classification identified all noises, including vocalizations and nonvocalizations. One researcher (N.H.P.) manually classified all auditory events as either nonvocalizations or calls from either Bird 1 or 2 (left or right channel, respectively). Auditory events were manually classified based on visual assessment of the spectrogram and time waveform and assigned to the appropriate channel based on the amplitude of the signal on the time waveform. While call types were not distinguished in the current dataset, the large majority of calls produced were stack calls (with some distance calls). Stack calls are the most common call type used in this behavioral assay (Prior et al. 2019), are commonly used between mates outside of a breeding context (Gill et al. 2015; D’Amelio et al. 2017b; D'Amelio 2018), and encode information on sex and individual identity (D’Amelio et al. 2017a; Prior et al. 2018). Stack calls appear to be important for communicating information about movement of partners when they are separated by short distances (D’Amelio et al. 2017b; D'Amelio 2018).

#### Operational definition of behavioral coordination

The coordination of movements and calling were quantified separately (Prior et al. 2020). First, the similarity in activity rates within a dyad was calculated (Similarity of Calling = (Call Rate of Bird 1–Call Rate of Bird 2)/Call Rate of Bird 1). Second, the temporal synchronization of social dynamics was estimated using sliding correlation coefficients (based on Pearson correlations) of the time-stamped list of movements and events that was generated for each recording. More specifically, the sliding correlation coefficients were calculated separately within the vocalizations and movements using the MATLAB “corrcoef” function. The step size for the sliding correlations was chosen based on the natural temporal dynamics of the movements and calls which we assessed during preliminary observations and development of the programs. For calculation of calling synchrony, a 1 ms sliding correlation timestep was used. For calculation of movement synchrony, a 40 ms sliding correlation timestep was used. Inputs to the sliding correlation computations were two vectors of ones and zeros, with a one indicating presence of a movement or call during the sliding window (1 ms for calls and 40 ms for movements) and a zero indicating absence of a movement or call. The sensor signal power in each time step was computed. For statistical analyses, we used the maximum Pearson’s correlation coefficient value (“corrcoef”) based on all possible temporal offsets.

#### Disentangling activity and coordination for calls and movements

The above approaches allowed us to quantify two crucial components of the social interaction: activity and coordination. However, we would predict that social interactions are inherently multimodal, and that the amount of activity and the coordination of activity might be related. Therefore, we used PCAs to describe the interrelationships between the four dependent variables (call rate, movement rate, sliding correlation of time-stamped calls, and sliding correlation of time-stamped movements). We conducted two separate PCAs, one for female and one for male (function “prcomp”) because there was a significant effect of sex on activity (see Results section).

For both females and males, the PCAs allowed us to successfully disentangle activity and coordination, and demonstrated the relationship between calling and physical behavior (Table 1). For both males and females, PC1 described overall activity (with call rate and movement rate being positively related), whereas PC2 describes coordination of activities with a positive relationship between the coordination of calling and the coordination of movements (Table 1: the first two components explained 67% and 74% and of the behavioral variation for females and males, respectively).

**Table 1:** PC loadings from the PCA analyses for females and males separately

	Female		Male	
	PC 1	PC 2	PC 1	PC 2
Cumulative variance (%)	36	67	46	74
Call rate	**−0.63**	0.44	**−0.67**	0.19
Movement rate	**−0.73**	−0.02	**−0.64**	0.23
Calling correlation coefficient	0.19	**0.66**	**−**0.37	**−0.50**
Movement correlation coefficient	0.23	**0.61**	0.10	**−0.81**

We considered parameters that loaded on their respective components >0.50 to be strong descriptors (bolded).

### Statistical analysis

All statistical analyses were carried out in R version 3.2.3, R Foundation for Statistical Computing. We used linear-mixed models (function lmer from the lme4 package). For each model, prior to interpretation, we transformed data as necessary based on a visual assessment of the distribution of the residuals. All data presented in graphs are nontransformed.

The effect of pair bonding on activity levels (call rate and movement rate) of males and female partners during social reunions was assessed using linear-mixed models with Pairing Stage and Sex as fixed factors and Individual Identity (BirdID) as a random factor (CallRate∼Sex*Pairing Stage+[1|BirdID]). The effect of pair bonding on the coordination of activities was assessed using linear-mixed models with Pairing Stage as a fixed factor and Pair ID as a random factor (sliding correlation coefficient calling∼ Pairing Stage +  [1|PairID]). Similarly, the effect of pair bonding on multimodal principal components (PC1 = activity and PC2 = coordination) was assessed separately for males and females with Pairing Stage as a fixed factor and Bird ID as a random factor. As we described above, we distributed the nine social reunion recordings based on key stages or pair bonding, rather than evenly throughout the month. For that reason, we chose to use Pairing Stage rather than Date as our primary variable. However, as a double check, the significant main effects that we report here for Pairing Stage are also present if we use Date as the primary factor.

We also conducted linear mixed models to determine if there was an effect of Prior Experience or subsequent Breeding Success on social interaction. Again, Prior Experience or Breeding Success were fixed factors, and Pair or Individual Identity was a random factor (e.g., sliding correlation coefficient calling∼ Pairing* Prior Experience +  [1|PairID]). Figures are presented with mean ± standard error of the mean.

## Results

### Activity levels of females and males during social reunions

**Fig. 2:**
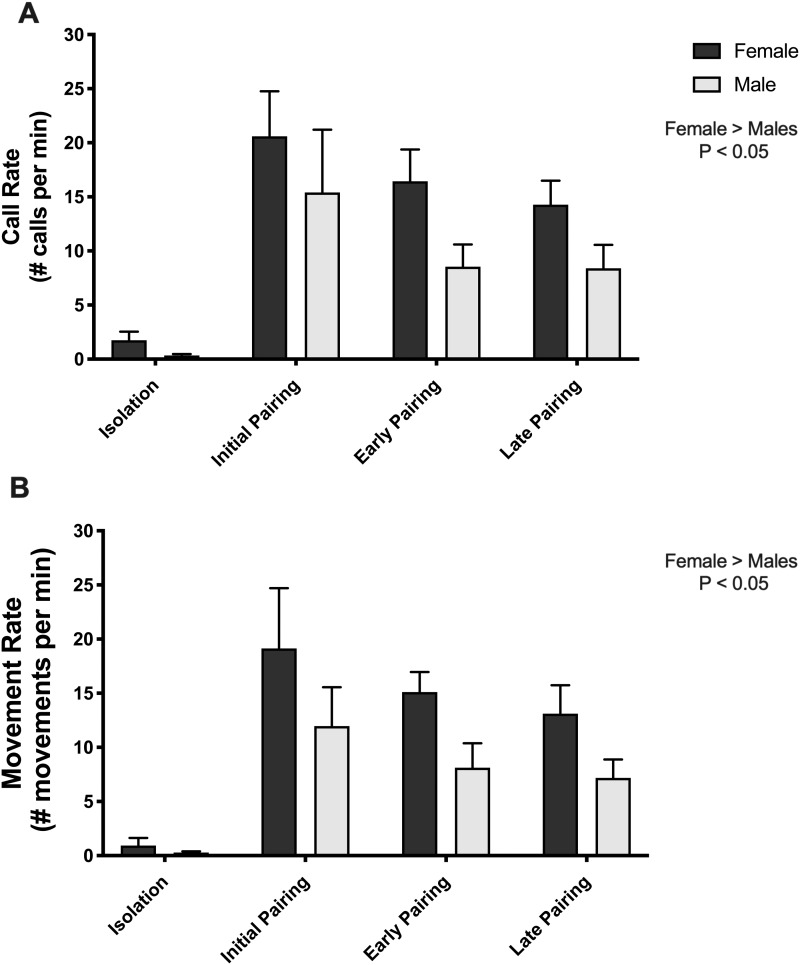
Overall activity rates, call rate (**A**) and movement rate (**B**), are shown for females and males tested in isolation and with their partner across the three pairing stages. Overall, females were more active than males (Call Rate *χ*^2^ (1) = 4.65, *P* = 0.031; Movement Rate *χ*^2^ (1) = 5.72, *P* = 0.017). Additionally, male and female call rates were highest during the initial pairing (Pairing Stage *χ* (2) = 8.35, *P* = 0.015; Pairing Stage × Sex *χ* (2) = 0.677, *P* = 0.713).

While isolated (recorded individually in the testing room), both males and females were largely inactive (Fig. 2). Eighteen out of the 20 individuals had a movement rate of <1/min (nine females and nine males) and 15 out of the 20 individuals had a call rate of <1/min (six females and nine males). We recorded each partner alone in the testing room as a control and it is not included in our statistical models: however, the low levels of activity during isolation emphasize the extent to which we are able to elicit behavior with a social reunion.

During the social reunions of partners, both call rate and movement rate were higher for females than males, regardless of the stage of pair bonding (Fig. 2. Call Rate *χ*^2^ (1) = 4.65, *P* = 0.031; Movement Rate *χ*^2^ (1) = 5.72, *P* = 0.017).

### Calling activity is modulated by pairing stage

For both males and females call rates were highest during initial pairing (Fig. 2. Pairing Stage *χ*^2^ (2) = 8.35, *P* = 0.015; Pairing Stage × Sex *χ*^2^ (2) = 0.677, *P* = 0.713). This main effect was driven by a difference between the initial pairing and the late stage of pairing (summary of the linear mixed model, LMER *t*-value = −1.971, *P* = 0.051). There was a similar pattern of decreased movement rate during later pairing, but this effect was not significant (Fig. 2. Pairing Stage *χ*^2^ (2) = 4.52, *P* = 0.104; Pairing Stage × Sex *χ*^2^ (2) = 0.30, *P* = 0.861). The results from our PCA analyses further support the interpretation that the effect of pairing stage was specific to call rate, not movement rate. For both males and females, PC1 represented a composite multimodal activity score (call rate and movement rate were positively correlated; see Materials and Methods section). There was no effect of pairing stage on PC1 for females (*χ*^2^ (2) = 4.21, *P* = 0.122) or males (*χ*^2^ (2) = 3.43, *P* = 0.180).

### Pairing stage has no effect on the coordination of activities

**Fig. 3:**
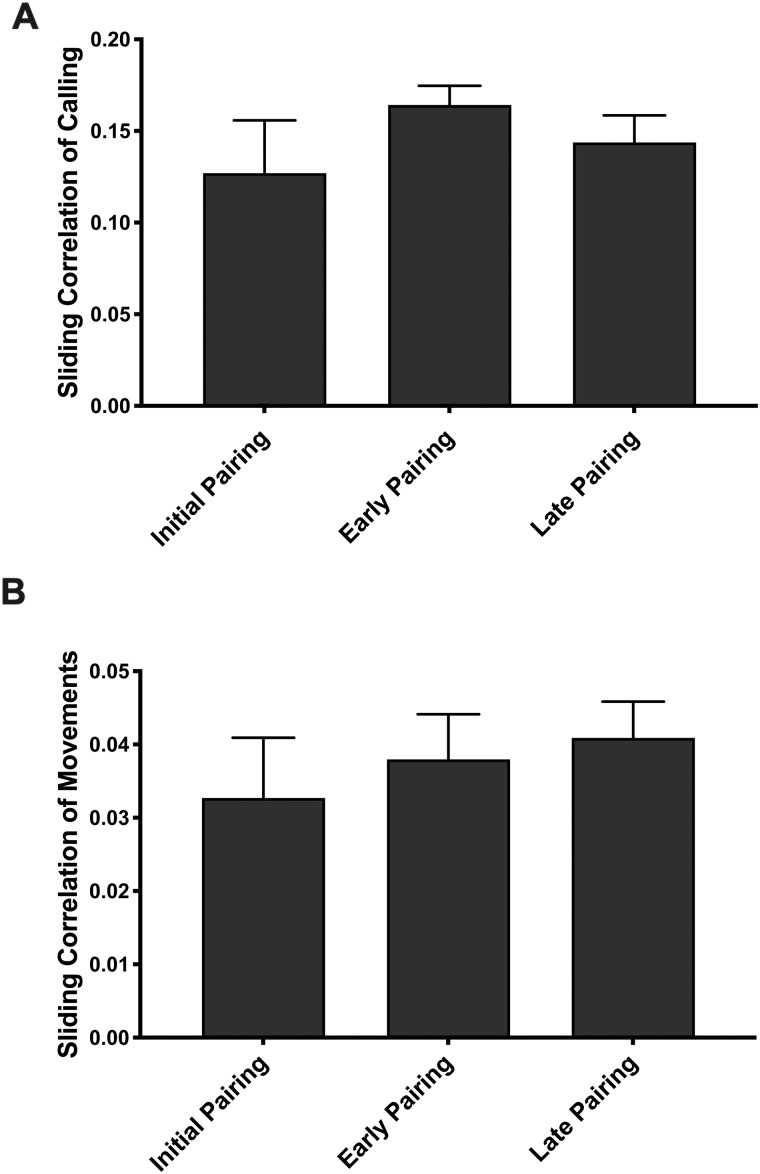
Pairing stage had no effect on the coordination of activities between males and females as quantified using the sliding correlation coefficient of time-stamped calls (**A**), and time-stamped movements (**B**), across the three pairing stages. As described in the text, there was also no effect of pairing stage on similarity of call rate and movement rate between partners.

There was no effect of pairing stage on coordination of activity for either calls or movements (Fig. 3). Pairing stage had no effect on the percent difference in call rate between female and male partners (F: M) (*χ*^2^ (2) = 1.47, *P* = 0.481), nor on the sliding correlation coefficient of calling activity (*χ*^2^ (2) = 2.85, *P* = 0.240). Likewise, there was no main effect of pairing stage on the coordination of movements (Percent Difference χ^2^ (2) = 0.11, *P* = 0.944; sliding correlation coefficient *χ*^2^ (2) = 1.00, *P* = 0.605). Again, the results of our PCA are consistent with the raw data. For both males and females PC2 represented a composite multimodal coordination score (the sliding correlation coefficient of calls and movements were positively correlated; see Materials and Methods section). There was no effect of pairing stage on PC2 for females (*χ*^2^ (2) = 1.09, *P* = 0.581) or males (*χ*^2^ (2) = 3.35, *P* = 0.187).

### Additional factors which may influence social reunion

**Fig. 4:**
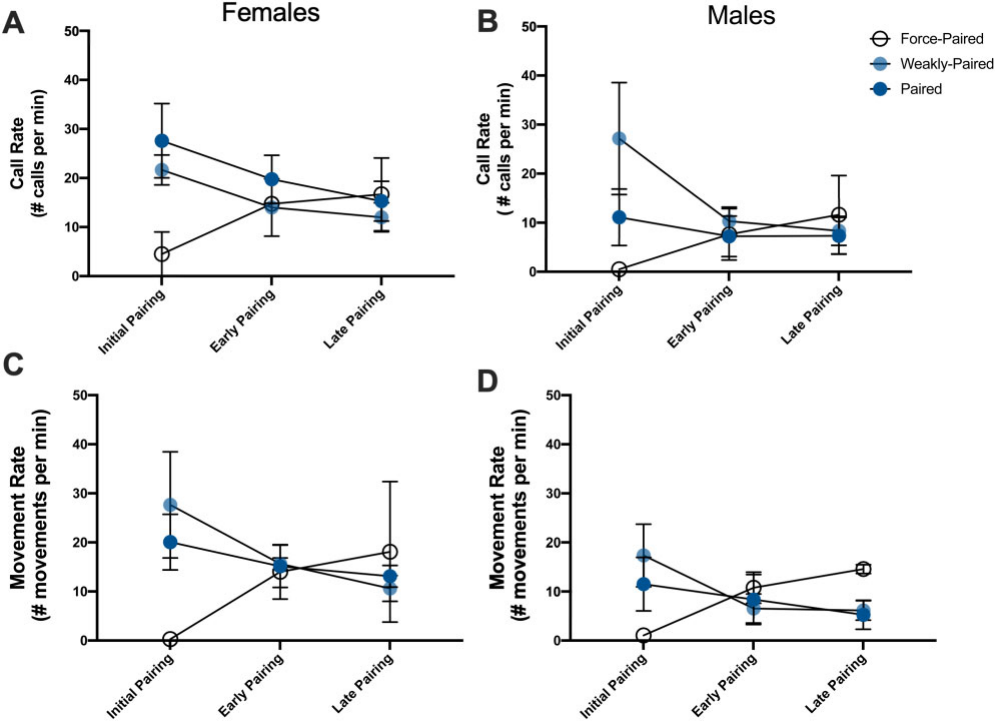
Effect of prior experience on calling activity (**A** and **B**) and movement rate (**C** and **D**) for females (**A** and **C**) and males (**B** and **D**) across the three stages of pairing. Pairs differed based on the amount of time and prior experience they had when they were initially paired after the 72 h being housed in mixed-sex flocks. Four pairs had clearly formed (paired). Another four pairs were created from individuals of the same mixed-sex flocks (weakly paired), with little evidence of pairing in the flocks. The final two pairs were composed of individuals that had no prior familiarity with each other (force paired). There was no effect of sex on either calling or movement, however, there was a significant interaction between Prior Experience and Pairing Stage (Calling: *χ* (4) = 17.44, *P* = 0.001; Movement: *χ* (4) = 17.88, *P* = 0.001).

Pairing stage was the primary factor we investigated. However, we had the opportunity to also ask whether there was the potential for a relationship between two additional features of the pairs and their behavior during the social reunion. First, we asked whether prior experience at the time of pairing influenced behavioral coordination. Indeed, there was a significant interaction between Prior Experience and Pairing Stage on activity (Fig. 4A–D. Calling Prior Experience χ^2^ (2) = 0.10, *P* = 0.950; Pairing Stage × Prior Experience *χ*^2^ (4) = 17.44, *P* = 0.001; Movement: Prior Experience *χ*^2^ (2) = 0.20, *P* = 0.903; Pairing Stage × Prior Experience *χ*^2^ (4) = 17.88, *P* = 0.001). There was also a significant interaction between Prior Experience and Pairing Stage on calling synchrony (sliding correlation coefficient of calls: Prior Experience *χ*^2^ (2) = 3.31, *P* = 0.191; Pairing Stage × Prior Experience *χ*^2^ (4) = 17.93, *P* = 0.001). However, there was no effect of prior experience on any other measure of the coordination of activities (Percent Difference in Calling: Prior Experience *χ*^2^ (2) = 2.03, *P* = 0.363; Pairing Stage × Prior Experience *χ*^2^ (4) = 7.01, *P* = 0.135; Percent Difference in Movement: Prior Experience *χ*^2^ (2) = 1.16, *P* = 0.561; Pairing Stage × Prior Experience *χ*^2^ (4) = 2.57, *P* = 0.633; Sliding Correlation of Movements: Prior Experience *χ*^2^ (2) = 0.30, *P* = 0.863; Pairing Stage × Prior Experience *χ*^2^ (4) = 4.49, *P* = 0.383).

Second, we tested whether behavioral coordination in social interactions during early pairing corresponded with breeding success. After the conclusion of this study, pairs had multiple opportunities to breed together. In the first breeding attempt (birds were provided with nestboxes 2 months after the end of this experiment) all 10 pairs engaged in nest building behavior. During this first opportunity to breed, 7 out of the 10 pairs went on to successfully fledge chicks (between two and four chicks per clutch). Of the pairs that did not fledge chicks, one pair (a strongly paired dyad) built a nest but laid no eggs, while the two other pairs (one weakly paired and one force paired dyad) laid eggs that did not hatch. There was no difference in calling synchrony or movement synchrony between pairs that successful fledged chicks and those that did not (Sliding Correlation of Calls: *χ*^2^ (2) = 0.24, *P* = 0.621; Sliding Correlation of Movements: *χ*^2^ (2) < 0.01, *P* = 0.978).

## Discussion

Here we describe the subtle changes that occur during brief social reunions between monogamous partners over the first month of pairing. The primary effect was elevated calling of both the male and female partner during the initial social reunion stage compared to later stages. Interestingly, we saw no change in coordination of calls or movements over the course of pairing. This is particularly notable because we have demonstrated previously that social greets with novel conspecifics elicit less robust behavioral responses and that those interactions are less coordinated (Prior et al. 2020). Together this suggests that zebra finches quickly develop familiar patterns of interactions with conspecifics.

Beyond the primary effect of pairing on calling activity, this study raises additional questions about what factors influence the social reunion of partners. First, across pairing stages, there were differences between females and males in their respective contributions to the interaction. Overall, females were more active than males (having higher call rates and movement rates). Second, pairs differed in how long they had been paired and the nature of the courtship experience (prior experience). Our data suggest prior experience influences patterns of behavior (both activity and the coordination of activities) during the initial, but not subsequent reunions.

### Changes in pair-directed affiliative behavior over time

For species that maintain long-term social bonds, including humans, the ability to maintain such bonds is as important as the ability to form them. Importantly, there is evidence that the neurobiological mechanisms underlying the formation of pair bonds differ from mechanisms underlying maintenance of bonds (Aragona et al. 2006; Smiley et al. 2012; Prior and Soma 2015; Resendez et al. 2016). There are many highly-marked affiliative behaviors that are associated with pair bonding, which can be useful in some species for identifying the point when pair bonds become established. Research investigating the changes in pairing over time has tended to parse these periods of pair bonding into discrete stages. In species such as zebra finches, where a mating event may not be needed for pair bond formation and individuals may not form a strong partner preference, it is particularly challenging to distinguish discrete stages of pairing.

Whereas the presence of highly-marked affiliative behaviors (e.g., clumping, side-by-side perching facing the same direction, and allopreening [Black and Hulme 1996; Zann 1996; Reichard and Boesch 2003]) are associated with the establishment of a bond, it is less clear how these behaviors change over time following initial pair bond formation. There is some evidence from both prairie voles and zebra finches that even after pair bond establishment, pairs continue to increase the amount of time they spend in close proximity (D’Amelio et al. 2017b; Scribner et al. 2020).

In zebra finches, D’Amelio et al. (2017b) described the changes in social dynamics of new versus established zebra finch pairs in the home cage over a week. On the first day, newly paired zebra finches spent significantly less time in physical proximity (clumping) compared to the established pairs, but this difference between new and established pairs was almost negligible by the third day of pairing. This timeline is consistent with our current results and suggests that the most significant changes in social interactions between partners occur during the first few days of pairing. However, it is notable that the trend for a difference between new and established pairs described by [Bibr obaa034-B11][Bibr obaa034-B11][Bibr obaa034-B12]) was present throughout the week of recording. Similarly, in prairie voles time spent in close proximity between partners appears to increase over the first month of pairing (Scribner et al. 2020).

D’Amelio et al. (2017 b) also carefully described the effect of pair bonding on vocal interactions of new and established zebra finch pairs using continuous vocal recordings. On the first day of recording, newly paired individuals called less than predicted. Additionally, over the first week of pairing, calling dynamics between partners became more symmetrical: shifting from a scenario where one bird called more and the other answered to a scenario where each partner called and answered similarly. Importantly, even newly paired birds were clearly motivated to engage in call-response. It is striking that in our study such brief social interactions also seemed affected by prior experience and pairing stage. Here we report low calling for the force-paired dyads on the initial pairing recording; but overall saw elevated activity earlier. Combined, our current work and [Bibr obaa034-B11][Bibr obaa034-B12]) suggest that the pattern and nature of social interactions between partners is dynamical emerging during the first several days of pairing. Interestingly, the types of calling exchanges we elicited here are very similar to those elicited by [Bibr obaa034-B11][Bibr obaa034-B12]), and are predominately made up of stack–stack calls and responses. This highlights the ethological relevance of our current social reunion test. This study combined with recent research highlights the need for a more comprehensive description of the subtle ways that social dynamics of partners change over time.

### Monogamy and moment-to-moment behavioral coordination

Broadly, behavioral coordination across timescales is associated with gregariousness (Conradt and Roper 2005; Focardi and Pecchioli 2005) and is thought to increase social cohesion (Pays et al. 2007; King and Cowlishaw 2009) and affiliative behavior (Sakai et al. 2010). Furthermore, behavioral coordination between two individuals has been shown to promote prosocial behavior (Van Baaren et al. 2004; Ashton–James et al. 2007; Gueguen et al. 2009) (reviewed in [Duranton and Gaunet 2016]), which we would expect to reflect the presence of affiliative bonds. Even on an extremely acute timescale, such as that examined here, there is evidence in humans that social or interactional synchrony promotes the formation and reinforcement of affiliative bonds (Feldman 2007; Feldman and Eidelman 2007; Feldman et al. 2011). In songbirds specifically, there has been extensive research investigating the function of behavioral coordination in monogamous pairs particularly as it relates to breeding success and the coordination of biparental care (Mariette and Griffith 2012, 2015; van Rooij and Griffith 2013; Boucaud et al. 2016b). Interestingly, brief social interactions at the nest, only a few minutes long, appear to be essential for the coordination of parental duties across many species (reviewed by Prior 2020). In zebra finches, there are several lines of evidence demonstrating that parental duties are actively coordinated during interactive calling exchanges (Elie et al. 2010; Boucaud et al. 2016a; Villain et al. 2016; Boucaud et al. 2017). During incubation, female calling behavior predicts whether or not the male will relieve her and begin incubation himself (Boucaud et al. 2017). Experimentally preventing the male from returning to the nest to relieve the female causes her to modify her calling behavior, which reflects her subsequent parental behavior (the amount of time she takes away from the nest) (Boucaud et al. 2016b). Additionally, experimental evidence from other avian species suggests that the coordination of parental behavior is an emergent consequence of the behavioral interactions between partners, not simply a summation of both partners’ contributions (Ball and Silver 1983). How partners develop such patterns of communication and what makes partners good communicators remains an open question.

It is possible that the patterns of a partner’s communication during mundane social interactions lays the foundation for the more salient or marked moments typically associated with social bonding. After the end of our current experiment, all the pairs were given the opportunity to breed. During this first breeding opportunity, 7 out of 10 pairs eventually went on to successfully fledge chicks, although there were no differences in behavioral coordination of the pairs that successfully fledged chicks and those that did not. The fact that we saw no relationship between behavioral coordination and breeding success is not surprising given the low number of pairs examined as well as the amount of time between the last social reunion recording and subsequent breeding. Furthermore, previous research has not suggested that temporal synchrony, independent of other characteristics, is the most important aspect of these social interactions. Future research will directly test the relationship between moment-to-moment coordination of activities and the coordination of parental behavior in order to further elucidate the function of behavioral variation in these brief interactions.

### Effect of familiarity and prior experience on social reunions

An intriguing potential confound exists when examining the long-term effects of pair bonds on social dynamics: to what extent can the nature of the social relationship (a monogamous pair bond) be disentangled from familiarity and shared social experience. Using the same social reunion behavioral assay, we recently demonstrated that familiarity itself, not pair bonding *per se*, influences social reunion behavior (Prior et al. 2020). When we compared reunion behavior between different social dyads (monogamous partners, familiar same-sex dyads, familiar opposite-sex dyads, novel same-sex dyads, and novel opposite-sex dyads), we found that both activity levels and the coordination of activity was higher in familiar social dyads (Prior et al. 2020). In our study, only the two force paired dyads (paired for ∼4 h) were barely active at all, similar to the novel dyads described previously. Thus, we can assume that pairs were able to familiarize and stabilize their degree of coordination very quickly, between 4 and 72 h. Combined, we interpret the results of these two studies as demonstrating an effect of prior social experience on behavioral coordination and patterns of behavior during brief social interactions.

Over the course of pair bonding, the fact that coordination is maintained but the activity decreases could be evidence that the strength of established bonds is negatively correlated with the length of social exchanges required. Perhaps well-established partners require ever-briefer, and less intense, social exchanges. Perhaps it is less the extent of coordination, but rather the the effort it takes to coordinate that reflects pair bond strength (although prior experience had no lasting effect on behavioral profiles beyond the initial courtship timepoint). Despite behavioral coordination/interactional synchrony being influenced by social bonding; the processes by which this happens may be a shared biological foundation of social alignment, rather than a pair bonding process specifically.

### Conclusion 

Here we show one way that mundane social interactions are affected by pair bonding, likely via shared prior social experience. Broadly speaking, this is consistent with the idea that social relationships are a culmination of repeated social interactions between familiar individuals. Understanding how brief social exchanges are modulated by experience and social bonding may provide an entry point to describe the wide diversity social relationships.
